# Supplementary figures and images for: The S-phase-induced lncRNA SUNO1 promotes cell proliferation by controlling YAP1/Hippo signaling pathway
Source: eLife. 2020 Oct 27;9:e55102. doi: 10.7554/eLife.55102 (PMC7591261; doi:10.7554/eLife.55102)

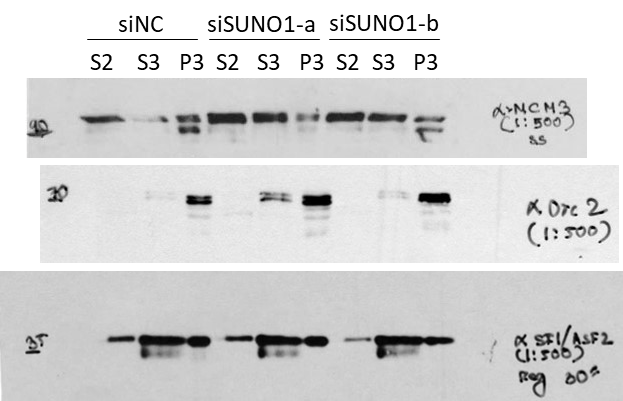
Figure 3E


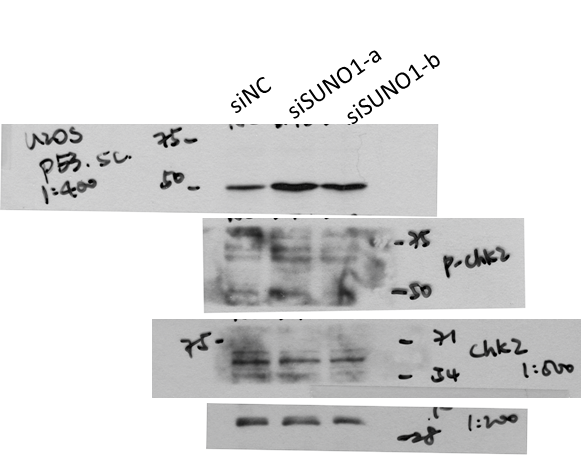


Figure 3-figure supplement 2C


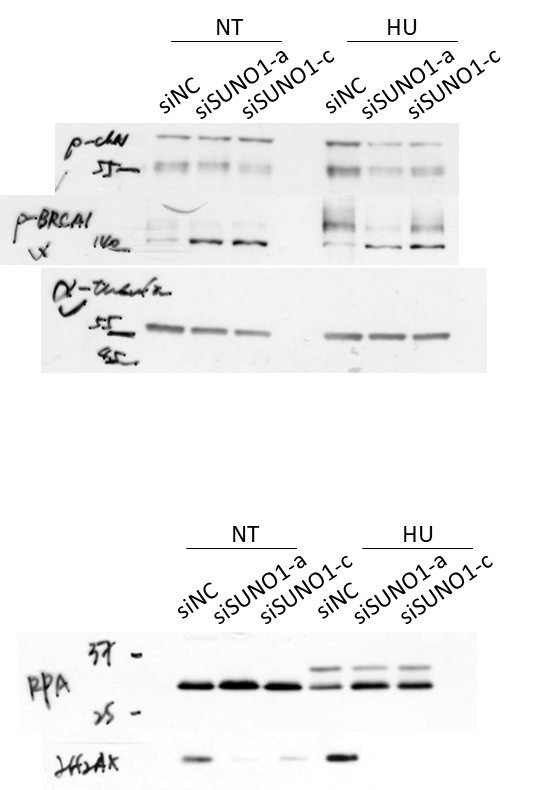

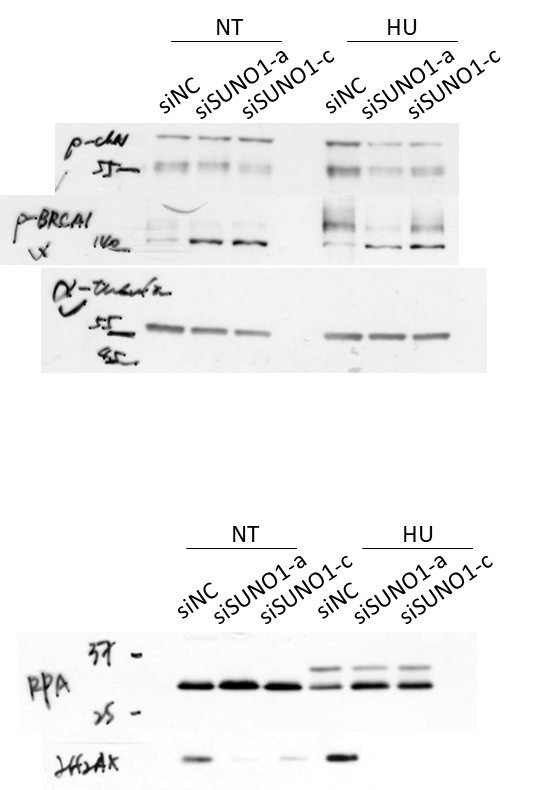
Figure 3-figure supplement 3B

Supplement: Figure 3—source data 1. [file elife-55102-fig3-data1.docx]

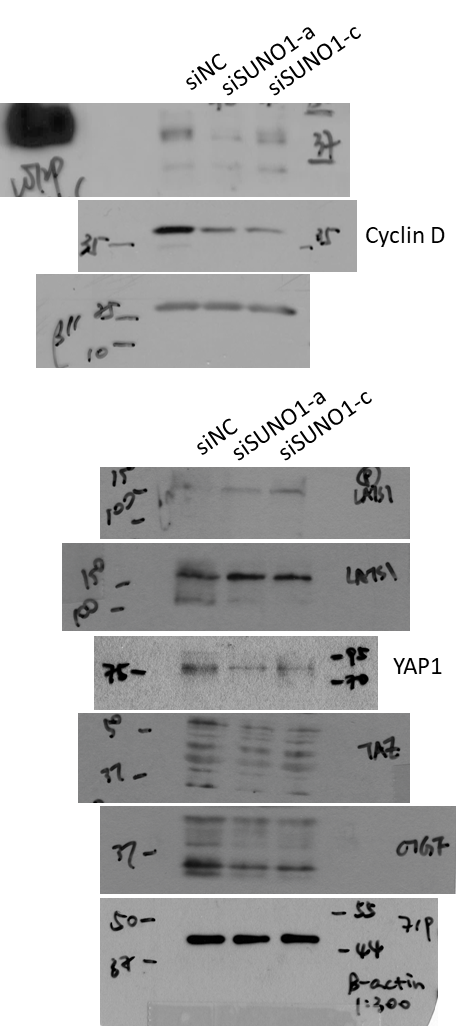

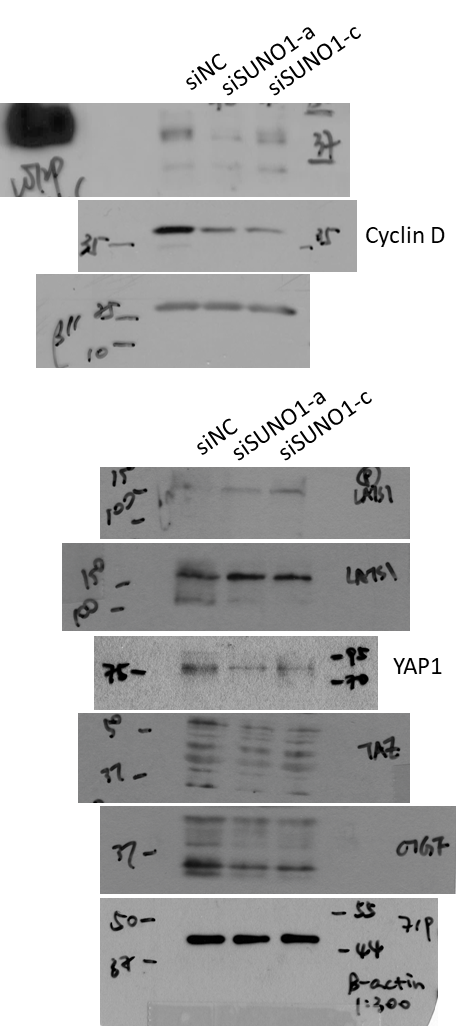
Figure 4C


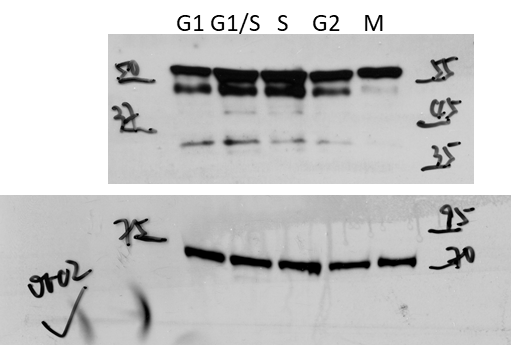
Figure 4-figure supplement 1F


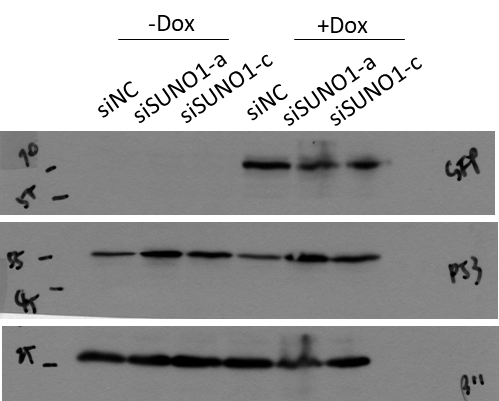
Figure 4-figure supplement 2A

Supplement: Figure 4—source data 1. [file elife-55102-fig4-data1.docx]
